# ﻿A new genus and five new species of pseudoscorpions (Arachnida, Pseudoscorpiones, Withiidae) from Colombia

**DOI:** 10.3897/zookeys.1184.106698

**Published:** 2023-11-22

**Authors:** Catalina Romero-Ortiz, Carlos E. Sarmiento, Mark S. Harvey

**Affiliations:** 1 Laboratorio de Sistemática y Biología Comparada de Insectos, Instituto de Ciencias Naturales, Universidad Nacional de Colombia, Bogotá, Colombia Universidad Nacional de Colombia Bogotá Colombia; 2 Collections and Research, Western Australian Museum, Welshpool, Western Australia 6106, Australia Western Australian Museum Welshpool Australia; 3 School of Biological Sciences, The University of Western Australia, Crawley, Western Australia 6009, Australia The University of Western Australia Crawley Australia

**Keywords:** Arachnids, bromeliads, Colombia, diversity, taxonomy

## Abstract

The pseudoscorpion family Withiidae is widely distributed around the world, with most of its diversity in tropical areas. Five new species and a new genus from Colombia are described: *Cystowithiusflorezi***sp. nov.**, *Parawithiusbromelicola***sp. nov.**, *Oligowithiusachagua***sp. nov.**, and the genus *Paciwithius***gen. nov.** with two species *Paciwithiusvalduparensis***sp. nov.** and *Paciwithiuschimbilacus***sp. nov.** A reassessment of the subgenus Dolichowithius (Oligowithius) Beier, 1936 allows the elevation to a full generic level, and the transfer of the only known species to *Oligowithius*, forming the new combination *Oligowithiusabnormis* (Beier, 1936), **comb. nov.**

## ﻿Introduction

With a surface area of more than 1.1 million square kilometers, Colombia is considered a hot-spot of biodiversity with nearly 2,000 species of birds (ACO 2020), 1,610 fish (DoNascimiento et al. 2019), 543 mammals (SCmas 2021), and 27,713 plants and lichens (Bernal et al. 2020). However, the Colombian pseudoscorpion fauna is rather poorly known even though this has been steadily rising during the past decade, with 23 recorded species in 2007 (Ceballos and Florez 2007) to 65 species in 2019 (Romero-Ortiz et al. 2019). But when compared with countries like Brazil with 174 species, Peru with 45 species, Ecuador with 61 species, and Venezuela with 63 species, there are considerable gaps to fill.

The pseudoscorpion family Withiidae Chamberlin, 1931 comprises more than 170 species arranged in 37 genera. The family is divided into two subfamilies, Paragoniochernetinae and Withiinae, with the latter divided into four tribes, Cacodemoniini, Juxtacheliferini, Protocheliferini, and Withiini. The status of these groups is highly uncertain, with only the Cacodemoniini seemingly supported by a strong synapomorphy in the male genitalia (Harvey 2015). Withiidae has a cosmopolitan distribution with its highest diversity in the Tropics. For the Neotropical region, there are ten genera recorded, six of them in the Cacodemoniini. Most of the taxonomic work on Neotropical withiids was undertaken in the 20^th^ century and accompanied by few illustrations. One of the most recent studies concerning New World withiids examined the status of the genera *Parawithius* Chamberlin, 1931 and *Victorwithius* Feio, 1944, and described a new genus *Cystowithius* Harvey, 2004 (Harvey 2004). Most recently, a new species of the genus *Cystowithius* was described (García and Romero-Ortiz 2021).

In this paper, we describe a new genus, *Paciwithius* gen. nov. with two new species, describe one new species of *Cystowithius*, register a new record of the species *Cystowithiusankeri* Garcia & Romero, 2021 and propose an identification key for the species of this genus, describe a new species of the genus *Parawithius*, and provide a reassessment of the subgenus Dolichowithius (Oligowithius) Beier, 1936.

## ﻿Materials and methods

The specimens examined for this study are lodged in the Arachnological collection at the Instituto de Ciencias Naturales – Universidad Nacional de Colombia (**ICN-APs**). Specimens were studied by preparing temporary slide mounts by immersion in 75% lactic acid at room temperature for one to several days and mounting them on microscope slides with 10-mm coverslips supported by small sections of 0.25-mm or 0.50-mm diameter nylon fishing line. Specimens were examined with an Olympus BH–2 compound microscope and illustrated with the aid of a drawing tube. Photographs of the body were taken with a Leica MC-170 HD digital camera attached to a Leica M205A stereomicroscope, and then stacked by the Leica Application Suite version 4.6.0, with the specimen submerged in KY® gel. For the SEM pictures, specimens were critically point dried and coated with gold as standard protocols and mounted in copper tape (Álvarez-Padilla and Hormiga 2007). Photographs were taken with a Hitachi S-4700 in the American Museum of Natural History (**AMNH**) and a Hitachi T3030 Plus in the Western Australian Museum (**WAM**).

Measurements were taken at the highest magnification possible using an ocular graticule, taken to the nearest 0.005 mm. After study, the specimens were rinsed in water and returned to vials with the other body parts submerged in 75% ethanol. Dissected portions were placed in small capillary tubes sealed with cotton on both ends. The type specimens are deposited in the Arachnology collection at the Instituto de Ciencias Naturales-Universidad Nacional de Colombia (**ICN-Aps**).

Terminology and measurements largely follow Chamberlin (1931b), except for the nomenclature of the pedipalps, legs, and, with some minor modifications, the terminology of the trichobothria where we follow Harvey (1992), chelicera (Harvey and Edward 2007; Judson 2007) and faces of the appendages (Harvey et al. 2012). Male genitalia terminology follows Romero-Ortiz and Sarmiento (2021).

The following abbreviations were used for the trichobothria of the movable finger ***b*** = basal; ***sb*** = sub-basal; ***st*** = sub-terminal; ***t*** = terminal. For the trichobothria of the fixed finger: ***eb*** = exterior basal; ***esb*** = exterior sub-basal; ***est*** = exterior sub-terminal; ***et*** = exterior terminal; ***ib*** = interior basal; ***isb*** = interior sub-basal; ***ist*** = interior sub-terminal; ***it*** = interior terminal. For the cheliceral setae: ***b*** = basal seta; ***es*** = exterior seta; ***is*** = interior seta; ***ls*** = laminal seta. For the abdominal setae: ***gls*** = glandular setae.

## ﻿Taxonomic account

### ﻿Family Withiidae Chamberlin, 1931

#### ﻿Genus Paciwithius gen. nov.

Taxon classificationAnimaliaPseudoscorpionesWithiidae

593C7885-29AA-5DCD-9E0F-563C8DF48911

https://zoobank.org/5AC62707-61F9-436E-A3DA-EACCA305DC6A

##### Type species.

*Paciwithiuschimbilacus* sp. nov.

##### Diagnosis.

*Paciwithius* can be distinguished from all other withiids by the absence of tergal keels, absence of eyespots, absence of a tactile seta on tarsus IV, the trichobothrium *it* is midway between *isb* and *et*, the presence of a patch of glandular setae on sternite VIII with an elongated shape, and the first three to six tergites are entire. The male genitalia have dorsal apodemes extending over its entire length, lateral apodemes extending over most of the ejaculatory canal atrium, ejaculatory canal curved dorsally in lateral view and lateral rods straight, extending beyond the lateral apodemes in dorsal view. This genus was mentioned as “nr. *Victorwithius*” in Romero-Ortiz and Sarmiento (2021).

##### Etymology.

The genus name refers to the Latin for peace, *pax*. Colombia has had a long history of violence and since the peace agreement in 2016, much effort has been put into making this country a peaceful land, including the Truth Commission (Comisión de la Verdad). Thanks to all the efforts for peace, we do believe that the change is inevitable. The name is masculine.

##### Remarks.

This new genus is placed within the Cacodemoniini as it has the triangular elongation made by the fusion of the dorsal and the lateral apodemes in the male genitalia that are characteristic of that tribe (Harvey 2015; Romero-Ortiz and Sarmiento 2021). The trichobothrial pattern has *it* and *isb* separated, and the only other Neotropical cacodemoniine genera with the same pattern are *Tropidowithius* Beier, 1955, *Balanowithius* Beier, 1959, and *Victorwithius* Feio, 1944. *Paciwithius* gen. nov. differs from males of *Victorwithius* by the patches of glandular setae on several sternites (restricted to sternite VIII in *Victorwithius*), and differs from *Tropidowithius*, *Balanowithius*, and *Victorwithius* by the lack of eyespots and the lack of a tarsal tactile seta, which are present in these three latter genera. The only other withiid genera that lack a tactile seta on tarsus IV are *Nannowithius* Beier, 1932 and *Termitowithius* Muchmore, 1990 (see Harvey 2015).

#### ﻿ Paciwithius chimbilacus sp. nov.

Taxon classificationAnimaliaPseudoscorpionesWithiidae

7BDEF788-51CA-50D3-98F2-94B7FC5064C2

https://zoobank.org/BDB0823D-51CB-4F25-8B1C-BF95D10D8F7D

**Figure 1.**
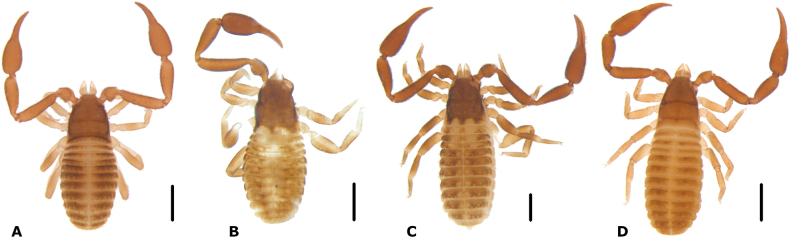
Habitus of the new species (males) **A***Paciwithiuschimbilacus* sp. nov. **B***Cystowithiusflorezi* sp. nov. **C***Parawithiusbromelicola* sp. nov. **D***Oligowithiusachagua* sp. nov. Scale bars: 0.5 mm.

**Figure 2.**
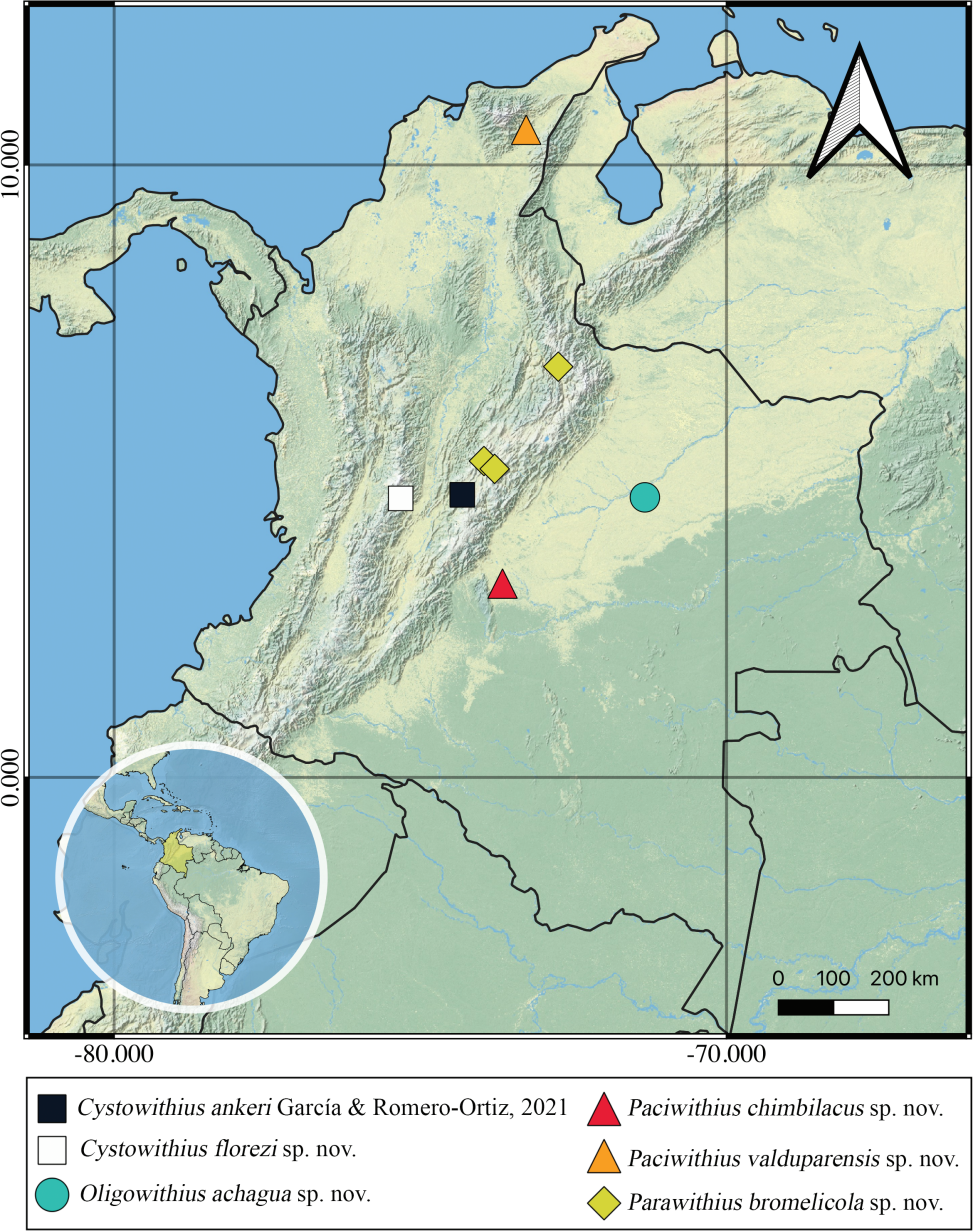
Distribution of the new species and the new record of Withiidae from Colombia.

**Figure 3.**
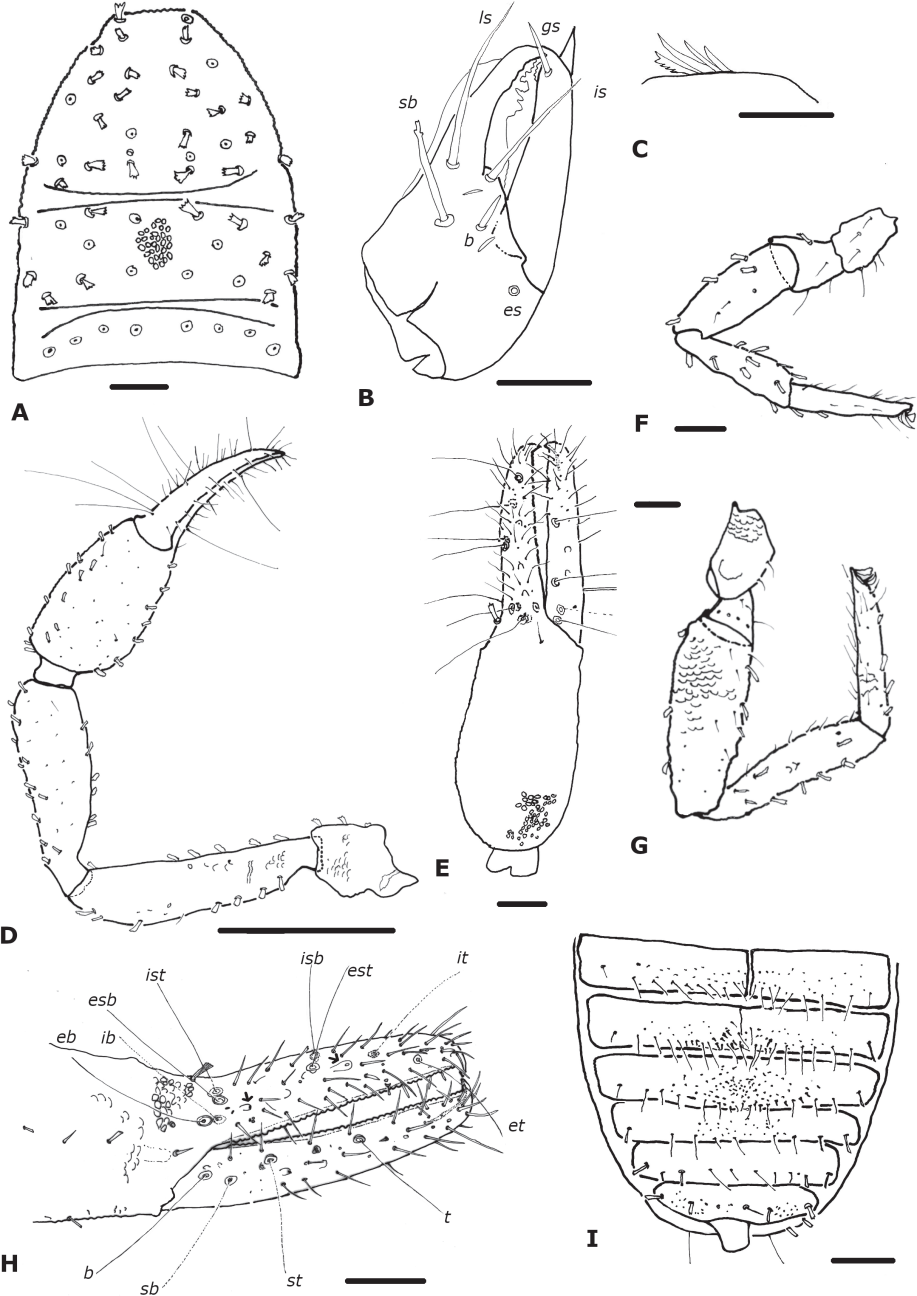
*Paciwithiuschimbilacus* sp. nov. (male holotype, ICN-Aps-076) **A** carapace **B** chelicera **C** rallum **D** left pedipalp **E** right chela **F** left leg I **G** left leg IV **H** right chela fingers (arrows show sense-spots) **I** abdomen, sternites. Abbreviations: Pedipalp movable finger: ***b*** = basal; ***sb*** = sub-basal; ***st*** = sub-terminal; ***t*** = terminal; fixed finger: ***eb*** = exterior basal; ***esb*** = exterior sub-basal; ***est*** = exterior sub-terminal; ***et*** = exterior terminal; ***ib*** = interior basal; ***isb*** = interior sub-basal; ***ist*** = interior sub-terminal; ***it*** = interior terminal. Cheliceral setae: ***b*** = basal seta; ***es*** = exterior seta; ***is*** = interior seta; ***ls*** = laminal seta. Scale bars: 0.5 mm (**D**); 0.1 mm (**A, E–I**); 0.05 mm (**B**); 0.0125 mm (**C**).

Figs 1A , 2 , 3 , 9A

##### Material examined.

***Holotype*.** Colombia • ♂; Meta, San Martín, Vda. San Francisco, Hacienda La María; [3°39'55.5"N, 73°39'29.7"W]; 400 m; on bat guano; ICN-APs-076.

***Paratype*.** Colombia • 1 ♂; same data as for the holotype; ICN-APs-076.

##### Diagnosis.

Males of *Paciwithiuschimbilacus* sp. nov. can be distinguished from *P.valduparensis* sp. nov. by a slender pedipalpal femur and patella (femur: 4.10–4.35× longer than broad in *P.chimbilacus* and 4.00 in *P.valduparensis*; patella: 3.33–3.43× longer than broad in *P.chimbilacus* and 2.27× in *P.valduparensis*), and a stouter chela (3.16–3.33× longer than broad in *P.chimbilacus* and 3.52× in *P.valduparensis*).

##### Description.

**Adults. *Color***: carapace brownish, darker than the tergites. Tergites yellow-brown, heavily granulated. Legs yellowish, proximal segments darker than the distal ones. Pedipalps reddish brown, heavily granulated; chela and fingers reddish (Fig. 1A).

***Dimensions*** (mm) (L/W): male: holotype: body length 1.93. Pedipalps: trochanter 0.34/0.20, femur 0.70/0.16, patella 0.63/0.18, chela (with pedicel) 0.94/0.26, chela (without pedicel) 0.88, hand (without pedicel) length 0.52, movable finger length 0.44. Chelicera 0.19, movable finger length 0.14. Carapace 0.66/0.52 (width at medial area). Leg I: femur 0.13/0.13, patella 0.27/0.11, tibia 0.29/0.08, tarsus 0.28/0.06. Leg IV: femur + patella 0.55/0.16, tibia 0.49/0.10, tarsus 0.37/0.06. Male: paratype: body length 1.78. Pedipalps: trochanter 0.36/0.19, femur 0.69/0.17, patella 0.64/0.19, chela (with pedicel) 0.92/0.27, chela (without pedicel) 0.86, hand (without pedicel) length 0.46, movable finger length 0.42.

***Carapace*** (Fig. 3A): 1.28× longer than broad; lateral margins not posteriorly widened; without eyes or eyespots; with 55 setae, distributed: 29 anterior (two near anterior margin), 18 in the mesozone, and eight near posterior margin, all clavate; with two distinct furrows; posterior furrow slightly closer to posterior carapace margin than to median furrow (Fig. 3A).

***Chelicera*** (Fig. 3B): with five setae on hand, *bs* denticulate; movable finger with single subdistal seta; galea simple; rallum of four blades, the most distal blade with several serrations on leading edge, other blades smooth (Fig. 3C); two dorsal lyrifissures.

***Pedipalp*** (Fig. 3D, E, H): trochanter, femur, patella and chelal hand granulate, fingers smooth; dorsal setae clavate; trochanter 1.72–1.88×, femur 4.10–4.35×, patella 3.33–3.43×, chela (with pedicel) 3.38–3.56×, chela (without pedicel) 3.16–3.33×, hand 1.69–1.97× longer than broad, movable finger 0.85–0.92× longer than hand. Fixed chelal finger with eight trichobothria, movable chelal finger with four trichobothria (Fig. 3E, H): *eb* and *esb* situated basally, as well as *ib* and *ist*; *isb* parallel to *est* both situated submedially, *it* midway between them and *et*; *b* and *sb* situated near one another, *st* closer to *sb* than to *t*, *t* situated submedially. Venom apparatus not visible, venom ducts not visible; nodus ramosus distal to *t* on movable finger, and not visible on fixed finger. Retrolateral margin of fixed finger with two sense-spots (Fig. 3H), one situated close to *esb* and *eb*, and the other distal to *est*. Chelal teeth squared; fixed finger with 35 teeth; movable finger with 37 teeth; accessory teeth absent.

***Coxal region***: coxal chaetotaxy: 20: 23: 24: 35; maxilla with 38 setae including two apical setae and one very small internal, sub-oral setae; median maxillary lyrifissure medial in position, posterior lyrifissure not visible.

***Legs*** (Fig. 3F, G): junction between femora and patellae I and II parallel, junction in legs III and IV oblique; tarsal tactile seta of leg IV absent; arolium slightly shorter than claws; claws simple; legs with scale-like appearance, many clavate setae on leg IV. Ratios: leg I: femur 1.00×, patella 2.43×, tibia 3.60×, tarsus 4.38× deeper than broad. Leg IV: femur + patella 0.33×, tibia 5.08×, tarsus 5.75× deeper than broad.

***Abdomen*** (Fig. 3I): tergites I–IV entire, the others with a faint medial suture, not keeled; sternites III–VII divided, sternites VIII–XI entire. Tergal chaetotaxy: 10: 10: 10: 11: 11: 12: 10: 12: 11: 8: 6: 4; mostly uniseriate but some tergites with a few setae placed anteriorly; all setae clavate. Sternal chaetotaxy: 10: (2) 13 (2): (2) 11 (2): 18: 19 + 1 *gls*: 20 + 24 *gls*: 13 + 84 *gls*: 9 + 4 *gls*+ 4 clavate: 10 + 7: 6 + 4 clavate setae: 2; sternites with many lyrifissures; sternite VI with one glandular seta, sternites VII–VIII of male with patches of glandular setae (Fig. 9A); glandular setae in extended patches (Fig. 9A); setae mostly uniseriate and acuminate but some clavate; male without paired invaginations on anterior margins of sternites.

***Genitalia***: see Romero-Ortiz and Sarmiento (2021) as “nr. *Victorwithius* 1”; male with elongated lateral apodemes although other structures not visible.

##### Etymology.

This species epithet is derived from the common name of bats in the Meta region “chimbilaco”, due to the bat guano where the specimens were found.

#### ﻿ Paciwithius valduparensis sp. nov.

Taxon classificationAnimaliaPseudoscorpionesWithiidae

9C04F161-97C1-5DEB-901E-44FF7007A0D8

https://zoobank.org/28B35490-FD34-4D13-855E-9F08C127BDFD

Figs 2 , 4 , 9C

##### Material examined.

***Holotype*.** Colombia • ♂; Cesar, Valledupar, Ecoparque Los Besotes, campamento base; [10°34'30.0"N, 73°16'19.8"W]; 600 m; 17 Jul. 2015; CarbioTeam leg.; manual capture; ICN-APs-597.

##### Diagnosis.

Males of *Paciwithiusvalduparensis* sp. nov. can be separated from *P.chimbilacus* sp. nov. by their stouter pedipalpal femur and patella (femur: 4.10–4.35× longer than broad in *P.chimbilacus* and 4.00× in *P.valduparensis*; patella: 3.33–3.43× longer than broad in *P.chimbilacus* and 2.27× in *P.valduparensis*), and a slender chela (3.16–3.33× longer than broad in *P.chimbilacus* and 3.52× in *P.valduparensis*).

##### Description.

**Adults. *Color***: yellowish brown, darker in carapace and tergites; carapace metazone without paired pale spots; pedipalps brownish, somewhat paler than body, fingers reddish; legs yellow-brown, uniform color; all specimen heavily granulated.

***Dimensions*** (mm): male: holotype: body length 1.84. Pedipalps: trochanter 0.28/0.18, femur 0.61/0.15, patella 0.60/0.26, chela (with pedicel) 0.94/0.26, chela (without pedicel) 0.90, hand (without pedicel) length 0.44, movable finger length 0.47. Chelicera 0.21, movable finger length 0.15. Carapace 0.66/0.52 (width at medial area). Leg I: femur 0.14/0.11, patella 0.24/0.10, tibia 0.27/0.07, tarsus 0.29/0.05. Leg IV: femur + patella 0.50/0.14, tibia 0.40/0.09, tarsus 0.35/0.05.

**Figure 4.**
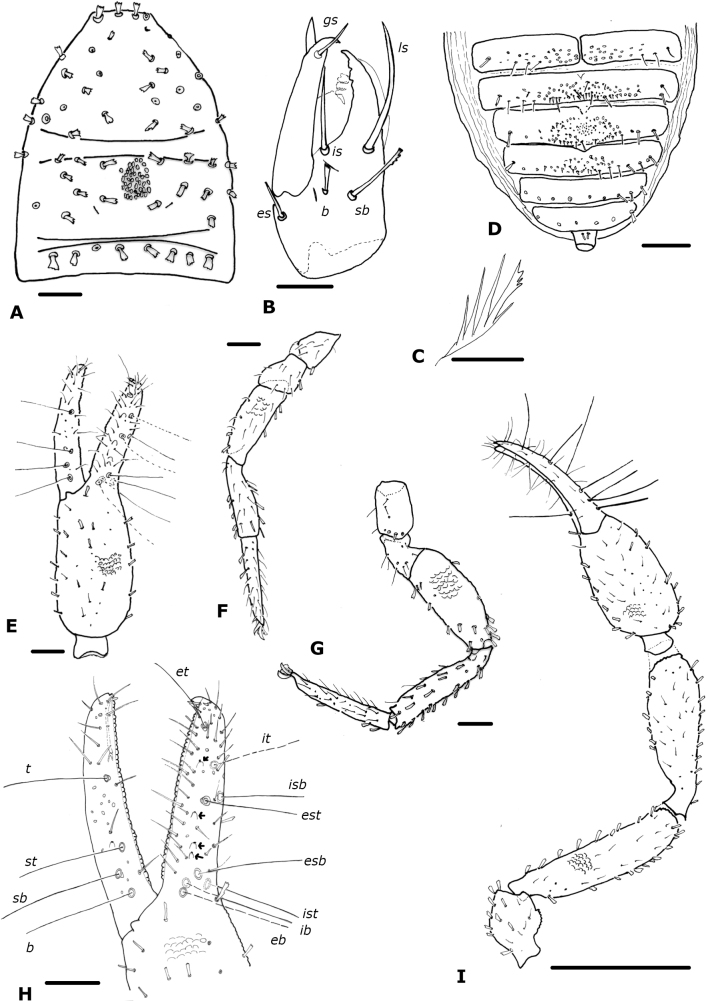
*Paciwithiusvalduparensis* sp. nov. (male holotype, ICN-Aps-597) **A** carapace **B** chelicera **C** rallum **D** abdomen, sternites **E** right chela **F** left leg I **G** left leg IV **H** right chelal fingers (arrows show sense-spots) **I** left pedipalp. Abbreviations: Pedipalp movable finger: ***b*** = basal; ***sb*** = sub-basal; ***st*** = sub-terminal; ***t*** = terminal; fixed finger: ***eb*** = exterior basal; ***esb*** = exterior sub-basal; ***est*** = exterior sub-terminal; ***et*** = exterior terminal; ***ib*** = interior basal; ***isb*** = interior sub-basal; ***ist*** = interior sub-terminal; ***it*** = interior terminal. Cheliceral setae: ***b*** = basal seta; ***es*** = exterior seta; ***is*** = interior seta; ***ls*** = laminal seta. Scale bars: 0.5 mm (**I**); 0.1 mm (**A, D–H**); 0.05 mm (**B**); 0.0125 mm (**C**).

***Carapace*** (Fig. 4A): 1.26× longer than broad; heavily granulated lateral margins convex, not posteriorly widened; without eyes; with 58 setae, distributed: 32 anterior (four near anterior margin), 18 in the mesozone, and eight near posterior margin, all clavate; with two distinct furrows; posterior furrow slightly closer to posterior carapace margin than to median furrow (Fig. 4A).

***Chelicera*** (Fig. 4B): with five setae on hand, *sb* and *b* denticulate, all others acuminate; movable finger with one subdistal seta; galea simple; rallum of four blades, the most distal blade with several serrations on leading edge, other blades smooth (Fig. 4C); two lyrifissures on dorsal and one on ventral side.

***Pedipalp*** (Fig. 4E, H, I): trochanter, femur, patella and chelal hand granulate, fingers smooth; dorsal setae clavate; trochanter 1.59×, femur 4.00×, patella 2.27×, chela (with pedicel) 3.67×, chela (without pedicel) 3.52×, hand 1.72× longer than broad, movable finger 1.07× longer than hand. Fixed chelal finger with eight trichobothria, movable chelal finger with four trichobothria (Fig. 4E, H): *eb* and *esb* situated basally, as well as *ib* and *ist*; *isb* parallel to *est* both situated submedially, *it* midway between them and *et*; *b* and *sb* situated near one another, *st* closer to *sb* than to *t*, *t* situated subdistally. Venom apparatus not visible, venom ducts not visible; nodus ramosus not visible. Retrolateral margin of fixed finger with four sense-spots (Fig. 4H), three situated linearly between *esb* and *est*, and the other between *est* and *et*. Chelal teeth squared; fixed finger with 42 teeth; movable finger with 42 teeth; accessory teeth absent.

***Coxal region***: coxal chaetotaxy: 10: 14: 15: 19; maxilla with 32 setae including two apical setae and one very small internal, sub-oral seta; median maxillary lyrifissure medial in position, posterior lyrifissure not visible.

***Legs*** (Fig. 4F, G): junction between femora and patellae I and II parallel, as well as legs III and IV; tarsal tactile seta of leg IV absent (Fig. 4G); arolium slightly shorter than claws; claws simple. Ratios: leg I: femur 1.29×, patella 2.31×, tibia 3.78×, tarsus 6.00× deeper than broad; Leg IV: femur + patella 3.44×, tibia 4.54×, tarsus 7.33× deeper than broad.

***Abdomen*** (Fig. 4D): tergites I–III, X, and XI entire, the others with faint medial suture, not keeled; sternites III–VI divided, entire from VII–XI. Tergal chaetotaxy: 10: 9: 9: 9: 12: 12: 12: 11: 12: 9: 4: 2; mostly uniseriate but some tergites with a few setae placed anteriorly; all setae clavate. Sternal chaetotaxy: 10: (2) 12 (2): (1) 6 (1): 19: 16 + 6 *gls*: 11 + 36 *gls*: 9 + 97 *gls*: 10 + 22 *gls*: 9: 6: 2; sternites with many lyrifissures, X–XII with lines of peak-like waves; sternites VI–IX with patches of glandular setae (Fig. 9C); glandular setae in extended patches (Fig. 9C); setae uniseriate and mostly acuminate, but some clavate in the lateral region of sternites VIII to XII; without paired invaginations on anterior margins of sternites.

***Genitalia***: see Romero-Ortiz and Sarmiento (2021) as “nr. *Victorwithius* msp. 2”.

##### Etymology.

This species is named after the city in which it was found. The Valledupar demonym is *valduparensis*. This is considered the place where the vallenato music was born. The specific epithet is an adjective.

#### ﻿Genus Cystowithius

Taxon classificationAnimaliaPseudoscorpionesWithiidae

Harvey, 2004

19EF5116-8941-5CF6-98D6-1E7094E9FF2D

Cystowithius Harvey 2004: 440; García and Romero-Ortiz 2021: 2.

##### Type species.

*Cystowithiussmithersi* Harvey, 2004, by original designation.

##### Diagnosis.

See Harvey (2004).

##### Remarks.

**Figure 5.**
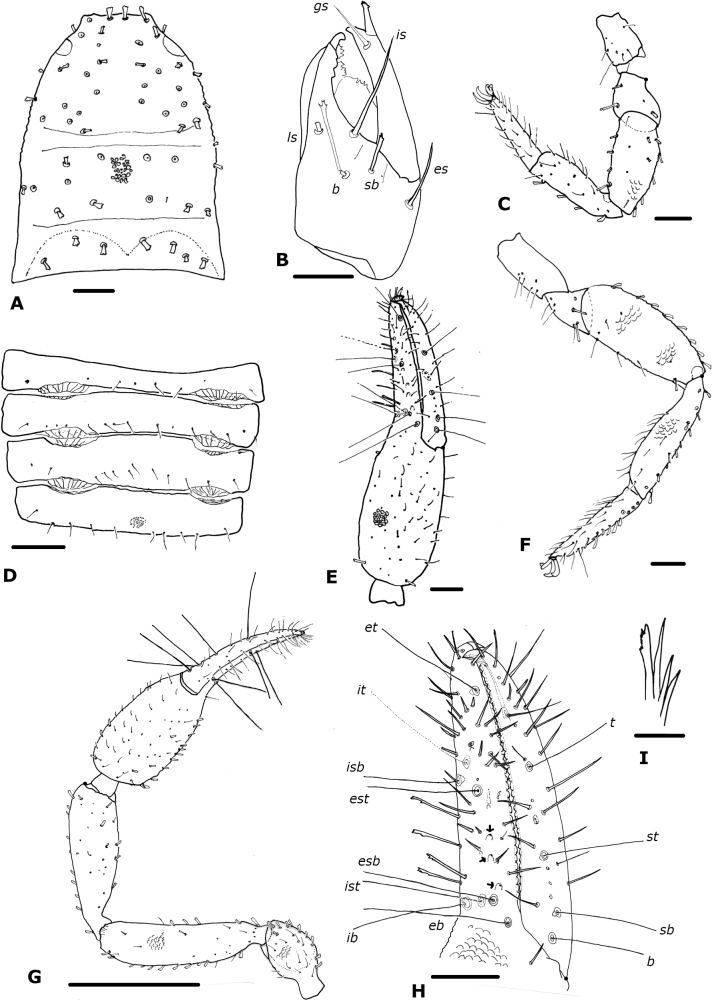
*Cystowithiusflorezi* sp. nov. (male holotype, ICN-Aps-077) **A** carapace **B** chelicera **C** left leg I **D** abdomen, sternites **E** right chela **F** left leg IV **G** left pedipalp **H** right chelal fingers (arrows show sense-spots) **I** rallum. Abbreviations: Pedipalp movable finger: ***b*** = basal; ***sb*** = sub-basal; ***st*** = sub-terminal; ***t*** = terminal; fixed finger: ***eb*** = exterior basal; ***esb*** = exterior sub-basal; ***est*** = exterior sub-terminal; ***et*** = exterior terminal; ***ib*** = interior basal; ***isb*** = interior sub-basal; ***ist*** = interior sub-terminal; ***it*** = interior terminal. Cheliceral setae: ***b*** = basal seta; ***es*** = exterior seta; ***is*** = interior seta; ***ls*** = laminal seta. Scale bars: 0.5 mm (**G**); 0.1 mm (**A, C–F, H**); 0.05 mm (**B**); 0.0125 mm (**I**).

**Figure 6.**
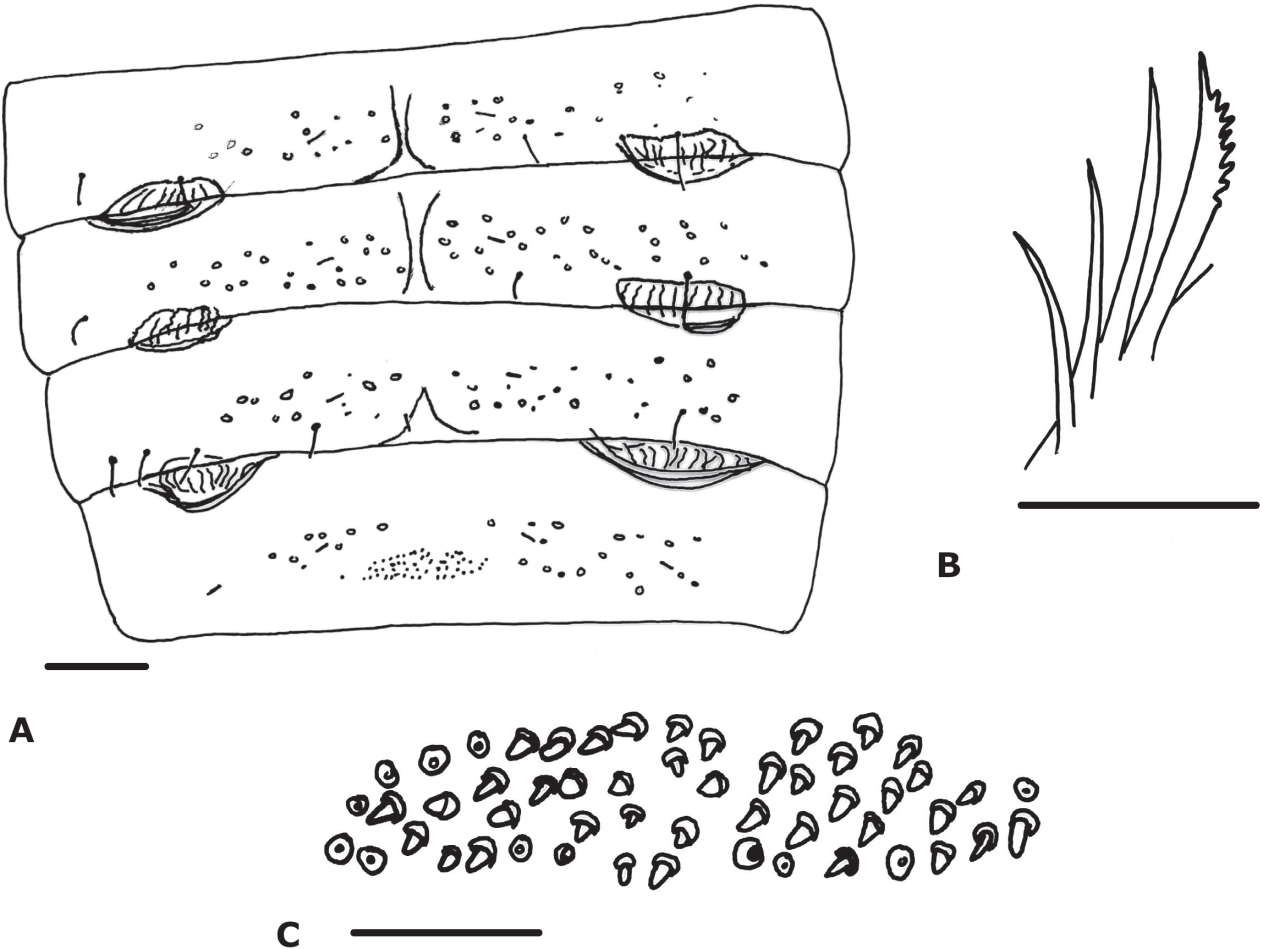
*Cystowithiusankeri* García & Romero-Ortiz, 2021 (male, ICN-Aps-298) **A** abdomen sternites **B** rallum **C** patch of glandular setae on sternite VIII. Scale bars: 0.1 mm (**A**); 0.0125 mm (**B, C**).

The genus *Cystowithius* is endemic to Central and South America (Harvey 2004; García and Romero-Ortiz 2021), and currently contains five species: *C.colombicus* Harvey, 2004 and *C.ankeri* García & Romero-Ortiz, 2021 from Colombia, *C.ecuadoricus* (Beier, 1959) and *C.smithersi* Harvey, 2004 from Ecuador and Peru, and *C.chamberlini* Harvey, 2004 from Mexico and Guatemala. Males are easily recognised by the presence of sternal invaginations (Figs 5D, 6A).

#### ﻿ Cystowithius florezi sp. nov.

Taxon classificationAnimaliaPseudoscorpionesWithiidae

710E2ECD-B1E1-5456-83F6-7D4D819225FC

https://zoobank.org/F2D766B6-215E-40D9-908B-76416F859F85

Figs 1B , 2 , 5 , 9D

##### Material examined.

***Holotype*.** Colombia • ♂; Tolima, Juntas, Reserva Natural Ibanasca; [4°33'22.0"N, 75°19'17.2"W]; 1700 m; 12 Feb. 2007; C. Cortes leg.; *Pinus* plantation; on low vegetation, manual capture; ICN-APs-077.

##### Diagnosis.

*Cystowithiusflorezi* can be separated from the other *Cystowithius* species as follows. *Cystowithiusflorezi* sp. nov. is very similar to *C.colombicus* Harvey, 2004 and *C.ecuadoricus* Harvey, 2004, so we provide a detailed comparison for each one in Table 1. In general, *C.colombicus* has bigger pedipalp segments and the patches of glandular setae located in sternites VII–IX; in *C.florezi* the patches are just in sternite VIII. Also, the sternal pockets are in sternites VI and VII in *C.colombicus* and in V–VIII in *C.florezi*. It differs from *C.chamberlini* by the chelal hand being smooth rather than strongly granulated in *C.florezi*, and the position of the tactile setae on tarsus IV located close to midway of the tarsus, rather than distal in *C.florezi*; from *C.ankeri* by the length of the movable chelal finger which is longer in *C.ankeri* than in *C.florezi* (0.70 mm vs 0.52 mm); and from *C.smithersi* by the length of the chela with pedicel which is longer in *C.smithersi* than in *C.florezi* (1.35 mm vs 1.02 mm).

**Table 1.** Comparative characters between three species of *Cystowithius*. **Bold** font denotes ratios.

Species/ Characters	*C.colombicus* Harvey, 2004	*C.ecuadoricus* Harvey, 2004	*C.florezi* sp. nov.
Body length	2.00–2.11	2.16–2.29	1.94
Carapace length/width	0.74/0.52	0.75–0.80/0.62	0.67/0.51
	**1.43**	**1.20–1.28**	**1.31**
Pedipalpal femur length/width	0.87–0.97/0.17–0.20	0.80–0.82/ 0.17–0.18	0.60/0.17
	**4.78–5.25**	**4.47–4.74**	**3.60**
Pedipalp patella length/width	0.77–0.88/0.19–0.21	0.67–0.69/0.20–0.21	0.61/0.18
	**3.88–4.46**	**3.18–3.47**	**3.30**
Chela with pedicel length/width	1.12–1.26/0.28–0.33	1.09–1.14/0.28–0.30	1.02/0.28
	**3.80–4.04**	**3.65–4.01**	**3.64**
Movable finger ratio (length/hand width)	0.49–0.61	0.50–0.56	0.52
	**0.90–1.24**	**0.86–1.11**	
Hand length/width	**0.50–0.60**	**0.50–0.58**	**0.49**
Femur/patella IV	0.67/0.17	0.60/0.21	0.57/0.18
		**2.93**	
Glandular setae	sternites VII–IX with patches of *gls* arranged ca. 20: 27: 13 respectively	sternites VI–IX with patches of *gls*, arranged 6: 42: 10: 8 (lectotype) respectively	sternite VIII with patches of *gls*, arranged 40 in a small circle
Sternal pockets on anterior margins of sternites	VI–VII	V–VIII	VI–VIII
Sense spots on chelal fingers	without sense spots on movable finger	without sense spots on movable finger but with small sense spot slightly distal to *st* that contains three small nubbins	without sense spots on movable finger but with small spot slightly distal to *st* that contains two small nubbins

##### Description.

***Color***: with sclerotized portions, generally yellow-brown; carapace and pedipalps darker; carapace metazone with paired pale spots; legs darker at the edges (Fig. 1B).

***Dimensions*** (mm): male: holotype: body length 1.94. Pedipalps: trochanter 0.36/0.18, femur 0.60/0.17, patella 0.61/0.18, chela (with pedicel) 1.02/0.28, chela (without pedicel) 0.96, hand (without pedicel) length 0.49, movable finger length 0.52. Chelicera 0.19, movable finger length 0.14. Carapace 0.67/0.51 (width at medial area); eye diameter 0.06. Leg I: femur 0.14/0.14, patella 0.29/0.14, tibia 0.20/0.10, tarsus 0.25/0.06. Leg IV: femur + patella 0.57/0.18, tibia 0.43/0.18, tarsus 0.34/0.07, TS 0.74.

***Carapace*** (Fig. 5A): 1.31× longer than broad; lateral margins convex, not posteriorly widened; with two non-corneate eyes; with 52 setae, including four near anterior margin, seven near posterior margin, 13 in the medial zone, and 32 in the anterior region; with two distinct furrows; posterior furrow slightly closer to posterior carapace margin than to median furrow (Fig. 5A); deeply granulated.

***Chelicera*** (Fig. 5B): with five setae on hand, *sb* and *b* denticulate, all others acuminate; movable finger with one subdistal seta; galea with one sub-terminal and three terminal rami; rallum of four blades, the most distal blade with several serrations on leading edge, other blades smooth (Fig. 5I); lamina exterior present; two dorsal lyrifissures.

***Pedipalp*** (Fig. 5E, G, H): trochanter, femur, patella and chelal hand granulate, chelal fingers smooth; setae clavate and denticulate; trochanter 2.04×, femur 3.81×, patella 3.30×, chela (with pedicel) 3.64×, chela (without pedicel) 3.42×, hand 1.74× longer than broad, movable finger 1.07× longer than hand. Fixed chelal finger with eight trichobothria, movable chelal finger with four trichobothria (Fig. 5E, H): *eb* and *esb* situated basally, as well as *ib* and *ist*; *isb*, *it* and *est* grouped together submedially; *et* near the distal end of the finger; *b* and *sb* situated near one another; *st* slightly closer to *sb* than to *t*; *t* parallel to *isb*. Venom apparatus not visible, venom ducts not visible in fixed finger; nodus ramosus distal to *t* on movable finger, not visible on fixed finger. Retrolateral margin of fixed finger with three sense-spots (Fig. 5H) situated linearly between *esb* and *est*; movable finger with small structure between *t* and *st* that contains two small nubbins. Chelal teeth rounded with an apical spot; fixed and movable finger with 32 teeth; accessory teeth absent.

***Coxal region***: coxal chaetotaxy: 8: 8: 8: 12, with multiple small lyrifissures; maxilla with two apical setae, one very small internal, sub-oral seta and 12 setae; median maxillary lyrifissure medial in position, posterior lyrifissure present.

***Legs*** (Fig. 5C, F): junction between femora and patellae I and II parallel, junction in legs III and IV oblique; tarsal tactile seta of leg IV situated distally, 0.74 of tarsus length (Fig. 5F); subterminal tarsal seta acute, distal to tactile seta; arolium same level as claws. Ratios: leg I: femur 1.06×, patella 2.12×, tibia 2.13×, tarsus 4.43× deeper than broad; Leg IV: femur + patella 3.09×, tibia 2.35×, tarsus 4.78× deeper than broad.

***Abdomen*** (Fig. 5D): all tergites divided but the first two only with a faint medial suture; all sternites entire. Tergal chaetotaxy: 8: 7: 8: 14: 15: 13: 15: 15: 15: 13: 8 (including two tactile setae): 2; mostly uniseriate but some tergites with a few setae placed anteriorly; all setae foliate, except in the last tergite; tergites densely granulated. Sternal chaetotaxy: 10: (2) 9 (2): (2) 9 (2): 12: 12: 12: 2 + 40 *gls*: 9: 8: 9 (including two tactile and three clavate setae): 2; only sternite VIII with a small circular patch of glandular setae (Fig. 9D); with paired invaginations on anterior margins of sternites VI–VIII (Fig. 5D).

***Genitalia***: see Romero-Ortiz and Sarmiento 2021 as “*Cystowithius* msp1”.

##### Etymology.

This species is dedicated to Professor Eduardo Florez, considered the father of Arachnology in Colombia. He has also been the curator of the Arachnological collection of the Instituto de Ciencias Naturales, where all the material used in this study is lodged.

#### ﻿ Cystowithius ankeri

Taxon classificationAnimaliaPseudoscorpionesWithiidae

Garcia & Romero-Ortiz, 2021

A576862B-679E-599E-903F-BE3FC41BBD1B

Figs 2 , 6

Cystowithius ankeri Garcia & Romero-Ortiz, 2021: 2–5, figs 1–12, 15.

##### Material examined.

Colombia • 3 ♂, 3 ♀; Cundinamarca, San Antonio del Tequendama, Parque Natural Chicaque; 04°36'51.3"N, 74°18'55.2"W; 2600 m; 1 Jun. 2009; F. Helbig leg.; on canopy log at 25 m height, manual capture; ICN-APs-298.

##### Remarks.

Although these specimens were collected 320 km from the type locality in Caldas Department, we refer them to the species *C.ankeri*. They have similar pedipalp proportions; however, their patches of glandular setae are different. The holotype of *C.ankeri* has two small patches in sternite VIII, and those examined here have one long patch of setae (Fig. 6A, C). We ascribe this characteristic to intraspecific variation.

### ﻿Key to the species of the genus *Cystowithius*

**Table:** 

1	Chelal hand smooth; setae on chelal hand only barely denticulate; tactile setae of tarsus IV situated closer to its mid-length (TS = 0.59–0.61 mm)	***C.chamberlini* Harvey, 2004**
–	Chelal hand evenly granulate; setae on chelal hand distinctly denticulate; tactile seta of tarsus IV situated subdistally (TS = 0.68–0.79 mm)	**2**
2	Movable chelal finger > 0.70 mm long; males with sternal invaginations on sternites VI–VIII	***C.ankeri* García & Romero-Ortiz, 2021**
–	Movable chelal finger < 0.60 mm long; males with sternal invaginations on sternites V–VIII or VI–VII	**3**
3	Pedipalps longer and more slender, i.e., chela (with pedicel) longer than 1.35 mm	***C.smithersi* Harvey, 2004**
–	Pedipalps shorter and more robust, i.e., chela (with pedicel) shorter than 1.20 mm	**4**
4	Femur of the pedipalp stout (3.96 times longer than broad), males with sternal invaginations on anterior margins of sternites VI–VIII, patches of glandular setae on sternite VIII in a small circle	***C.florezi* sp. nov.**
–	Femur of the pedipalp slender (> 4.47 times longer than broad), males with sternal invaginations on anterior margins of sternites VI–VIII, patches of glandular setae on sternite VIII in a small circle	**5**
5	Setae on tergite XI short and strongly clavate; chelal hand without long, strongly denticulate setae	***C.colombicus* Harvey, 2004**
–	Setae on tergite XI long and only slightly clavate; chelal hand with long, strongly denticulate setae	***C.ecuadoricus* (Beier, 1959)**

#### ﻿Genus Parawithius

Taxon classificationAnimaliaPseudoscorpionesWithiidae

Chamberlin, 1931

FEC9B230-5BFA-59C5-98C4-A5EA372A5B16

Parawithius Chamberlin, 1931a: 292; Beier 1932: 212; Beier 1959: 216; Harvey 2004: 437.

##### Type species.

*Chelifernobilis* With, 1908, by original designation.

##### Diagnosis.

See Harvey (2004).

##### Remarks.

Harvey (2004) delimited the genus *Parawithius* to include three South American species: *P.nobilis* (With, 1908) from Colombia, and *P.pseudorufus* Beier, 1932 and *P.iunctus* Beier, 1932 from Paraguay. We add a further species from Colombia below.

#### ﻿ Parawithius bromelicola sp. nov.

Taxon classificationAnimaliaPseudoscorpionesWithiidae

6062828A-DDB6-5E4B-BC56-5D0DBD10AC0E

https://zoobank.org/A1720398-A29E-4F9E-8E8E-6D9DD9EDFB71

**Figure 7.**
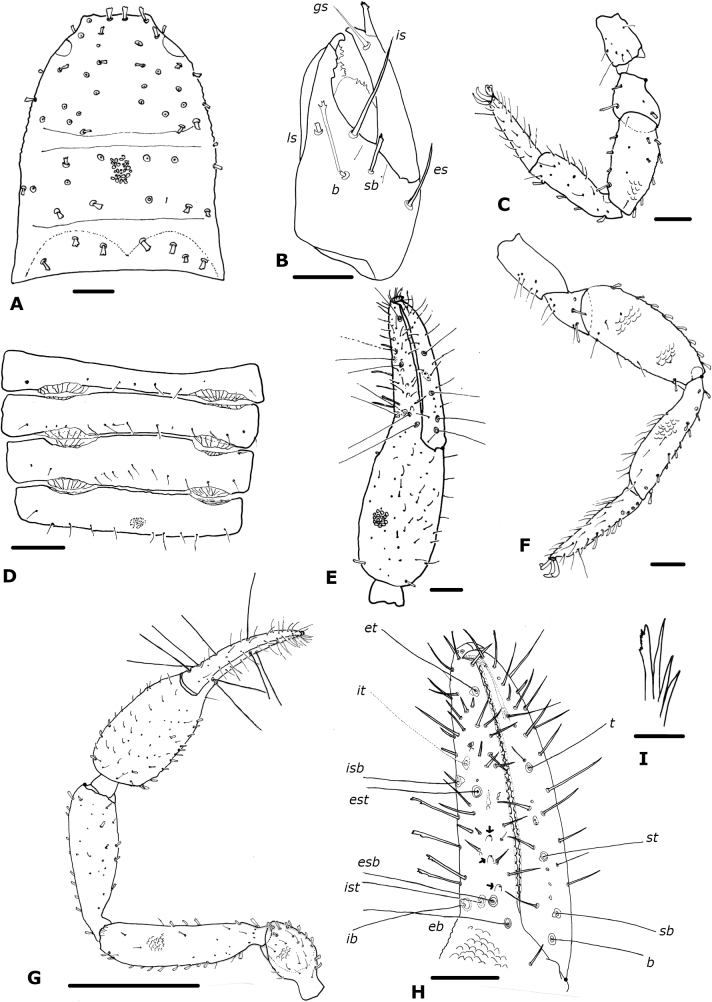
*Parawithiusbromelicola* sp. Nov. (male holotype, ICN-Aps-082) **A** carapace **B** chelicera **C** rallum **D** right chela **E** left leg I **F** left leg IV **G** left pedipalp **H** right chelal fingers (arrows show sense-spots) **I** rallum. Abbreviations: Pedipalp movable finger: ***b*** = basal; ***sb*** = sub-basal; ***st*** = sub-terminal; ***t*** = terminal; fixed finger: ***eb*** = exterior basal; ***esb*** = exterior sub-basal; ***est*** = exterior sub-terminal; ***et*** = exterior terminal; ***ib*** = interior basal; ***isb*** = interior sub-basal; ***ist*** = interior sub-terminal; ***it*** = interior terminal. Cheliceral setae: ***b*** = basal seta; ***es*** = exterior seta; ***is*** = interior seta; ***ls*** = laminal seta. Scale bars: 0.5 mm (**G**); 0.1 mm (**A, D–F, H**); 0.05 mm (**B**); 0.0125 mm (**C, I**).

Figs 1C , 2 , 7 , 9B

##### Material examined.

***Holotype*.** Colombia • ♂; Cundinamarca, Cogua, Embalse del Neusa Tausa, Llano Grande; [5°11'33.9"N, 73°53'53.8"W]; 2900 m; 7 Mar. 2004; AL Leon leg.; under tree bark; ICN-APs-082.

***Paratypes*.** Colombia • 1 ♂; Cundinamarca, same data as for the holotype. • 1 ♀, 1 ♂; Sesquilé, Camino al Cerro de Las Tres Viejas; 5°02'17.0"N, 73°47'13.0"W; 2740 m; 8 Sep. 2019; C. Romero-Ortiz, F. Garcia, J.J. Lagos, A. Carvajal, D. Mayorga-Ch leg.; on bromeliad; ICN-APs-836.

***Other material*.** Colombia • 1 ♀; Santander, Málaga, Vda. Buenavista, km 7 vía Bucaramanga; 6°42'23.7"N, 72°44'52.6"W; 2620 m; 1 Jan. 2020; C. Romero-Ortiz, J.J. Lagos leg.; on bromeliad under “Loqueto” tree *Escalloniapendula* (Ruiz & Pav.) Pers.; ICN-APs-847.

##### Diagnosis.

*Parawithiusbromelicola* sp. nov. can be separated from *P.nobilis* (With, 1908) by the stouter pedipalpal segments (i.e. patella 3.36–3.44× longer than broad compared with 3.24–3.30× longer than broad, and the chela without pedicel 3.39–3.47× longer than broad, compared to 3.58–3.85× longer than broad); the extension of the strongly clavate setae on the dorsal surface of fixed chelal finger (i.e., distal to *it* and *est* compared to proximal to *it* and *est*); from *P.iunctus* Beier, 1932 and *P.pseudorufus* Beier, 1932 by the presence of pale spots on the carapace metazone, in addition to the size of chelal fingers compared to the palpal hand (i.e. fingers shorter to the hand in *P.bromelicola* sp. nov., *P.nobilis*, and *P.iunctus* and longer in *P.pseudorufus*).

##### Description.

**Adults. *Color***: yellowish brown, carapace darker than body, carapace metazone with paired pale spots; pedipalps reddish brown, uniform in color, very granulated; tergites yellow-brown; big leg segments darker at posterior margin (Fig. 1C).

***Dimensions*** (mm): male: holotype (followed by male paratypes): body length 2.46 (2.58, 2.46). Pedipalps: trochanter 0.38/0.21 (0.37/0.23, 0.38/0.22), femur 0.83/0.19 (0.84/0.19, 0.77/0.18), patella 0.74/0.22 (0.75/0.22, 0.71/0.21), chela (with pedicel) 1.18/0.30 (1.24/0.31, 1.12/0.30), chela (without pedicel) 1.14 (1.18, 1.06), hand (without pedicel) length 0.59 (0.57, 0.53), movable finger length 0.56 (0.60, 0.53). Chelicera 0.21, movable finger length 0.17. Carapace 0.82/0.58 (0.85/0.72, 0.82/0.74); eyespot diameter 0.07. Leg I: femur 0.16/0.16, patella 0.33/0.14, tibia 0.35/0.10, tarsus 0.32/0.06. Leg IV: femur + patella 0.66/0.19, tibia 0.51/0.12, tarsus 0.37/0.07, TS 0.73.

**Female**: paratype (followed by female other material): body length 2.68 (2.70). Pedipalps: trochanter 0.41/0.23 (0.36/0.21), femur 0.78/0.21 (0.73/0.18), patella 0.74/0.23 (0.67/0.19), chela (with pedicel) 1.24/0.35 (1.14/0.30), chela (without pedicel) 1.16 (1.08), hand (without pedicel) length 0.59 (0.57), movable finger length 0.60 (0.49). Carapace 0.88/0.68 (0.74/0.64) (width at medial area).

***Carapace*** (Fig. 7A): 1.42× (1.18–1.11) (♂), 1.29× (1.16) (♀) × longer than broad; lateral margins convex, not posteriorly widened; with two non-corneate eyes; with 59 (♂) setae, including four (♂) near anterior margin, seven (♂) near posterior margin, 16 in the medial zone and 36 in the anterior region; with two distinct furrows; posterior furrow slightly closer to posterior carapace margin than to median furrow (Fig. 7A).

**Figure 8.**
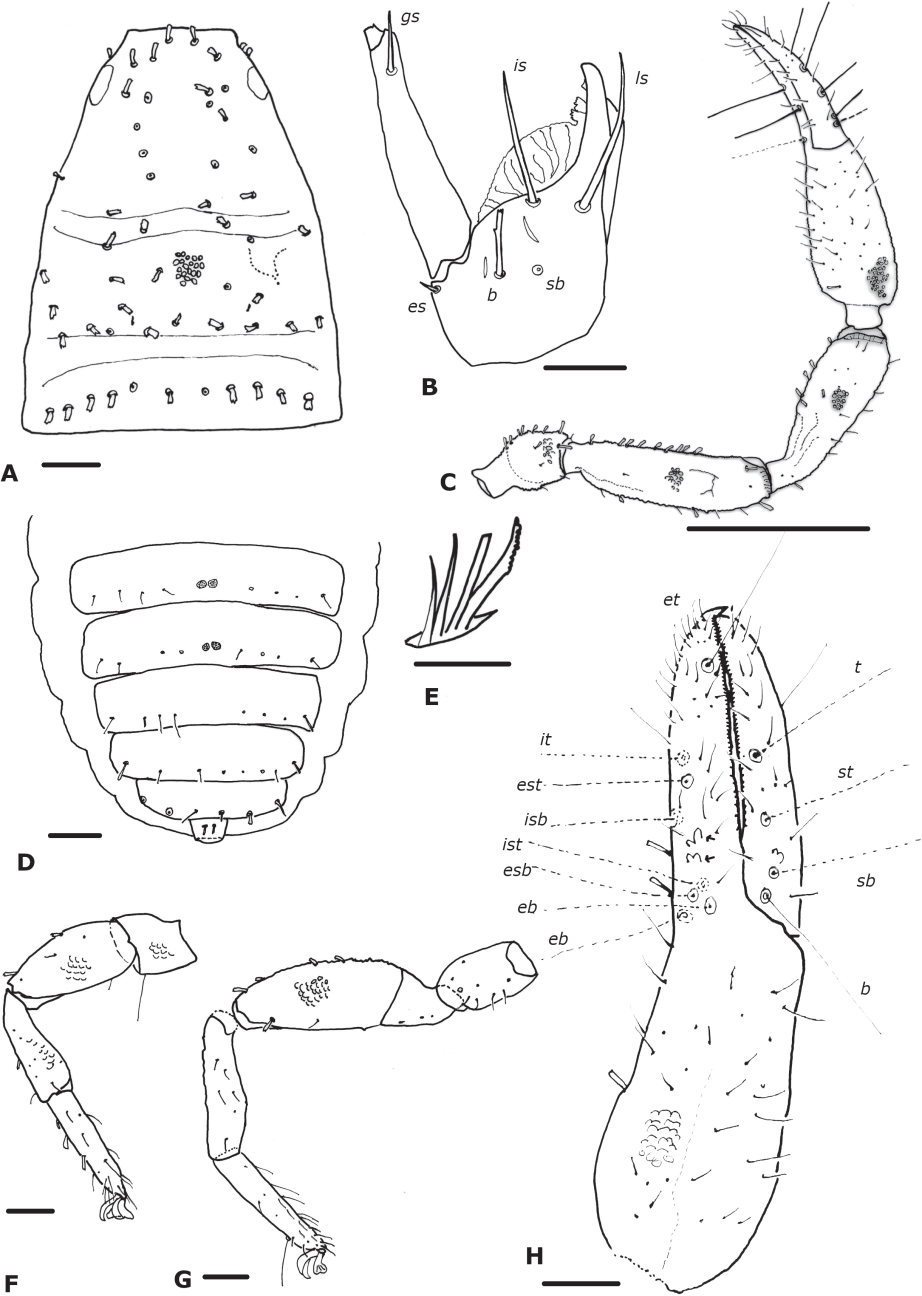
*Oligowithiusachagua* sp. nov. (male holotype, ICN-Aps-388) **A** carapace **B** chelicera **C** left pedipalp **D** abdomen, sternites **E** rallum **F** left leg I **G** left leg IV **H** right chela (arrow shows sense-spots). Abbreviations: Pedipalp movable finger: ***b*** = basal; ***sb*** = sub-basal; ***st*** = sub-terminal; ***t*** = terminal; fixed finger: ***eb*** = exterior basal; ***esb*** = exterior sub-basal; ***est*** = exterior sub-terminal; ***et*** = exterior terminal; ***ib*** = interior basal; ***isb*** = interior sub-basal; ***ist*** = interior sub-terminal; ***it*** = interior terminal. Cheliceral setae: ***b*** = basal seta; ***es*** = exterior seta; ***is*** = interior seta; ***ls*** = laminal seta. Scale bars: 8 µm (**A, C, D, F–H**); 2 µm (**B, E**). Granulation is shown in detail on each segment in a small section but is meant to cover all the structure.

***Chelicera*** (Fig. 7B): with five setae on hand, *sb* and *b* denticulate, all others acuminate; movable finger with one subdistal seta; galea of male with three or four very small terminal rami; rallum of four blades, the most distal blade with several serrations on leading edge, other blades smooth (Fig. 7C); two dorsal lyrifissures.

***Pedipalp*** (Fig. 7D, G, H): trochanter, femur, patella and chelal hand granulate, chelal fingers smooth; dorsal setae clavate and denticulate; trochanter 1.81× (1.59–1.74) (♂), 1.76× (1.73) (♀), femur 4.33× (4.17–4.38) (♂), 3.77× (4.14) (♀), patella 3.44× (3.36–3.42) (♂), 3.17× (3.50) (♀), chela (with pedicel) 3.99× (3.78–3.97) (♂), 3.52× (3.85) (♀), chela (without pedicel) 3.85× (3.58–3.78) (♂), 3.29× (3.65) (♀), hand 2.0× (1.78–1.82) (♂), 1.68× (1.92) (♀)longer than broad, movable finger 0.95× (1.00–1.06) (♂), 1.01× (0.86) (♀)longer than hand. Fixed chelal finger with eight trichobothria, movable chelal finger with four trichobothria (Fig. 7D, H): *eb* and *esb* situated basally, *est* midway between *eb* and the fingertip, *et* situated distally; *ib* and *ist* situated basally, *it* directly behind *est* and close distal to *isb*; *b* and *sb* situated near one another; *st* closer to *sb* than to *t*; *t* parallel to *est*. Venom apparatus not visible, venom ducts not visible in ♂ and ♀. Retrolateral margin of fixed finger with three sense-spots (Fig. 7H) situated distal to *esb* and linear in disposition. Chelal teeth very small and almost not developed or differentiated for a clear count; accessory teeth absent.

***Coxal region***: coxal chaetotaxy: ♂, 10: 10: 5: 7; maxilla with 23 setae including three apical setae (one tactile setae) and one very small internal, sub-oral seta; median maxillary lyrifissure medial in position, posterior lyrifissure present.

***Legs*** (Fig. 7E, F): junction between femora and patellae I and II parallel as well as junction in legs III and IV; tarsal tactile seta of leg IV situated distally, 0.73× (♂) of tarsus length (Fig. 7F); two subterminal tarsal setae distal form TS arcuate and acute; arolium shorter than claws. Ratios: leg I: femur 1×, patella 2.28×, tibia 3.67×, tarsus 5.71× deeper than broad; leg IV: femur + patella 3.46×, tibia 4.27×, tarsus 5.11× deeper than broad.

**Figure 9.**
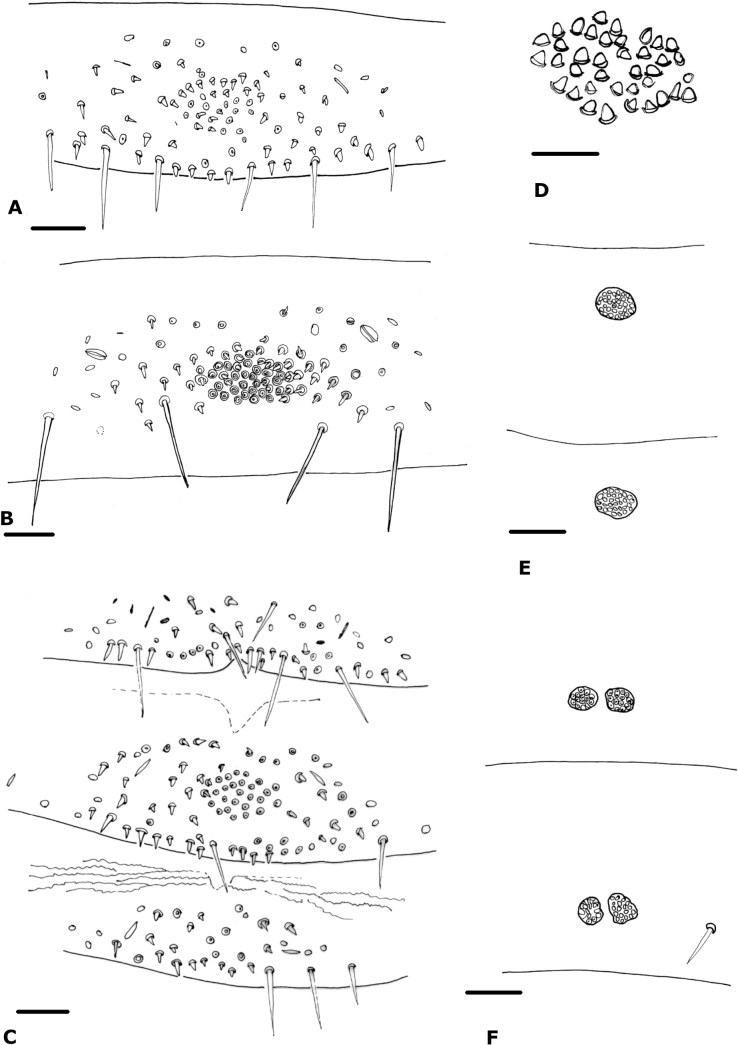
Glandular setae patches of the new species **A***Paciwithiuschimbilacus* sp. nov. (holotype, ICN-Aps-076) **B***Parawithiusbromelicola* sp. nov. (holotype, ICN-Aps-082) **C***Paciwithiusvalduparensis* sp. nov. (holotype, ICN-Aps-597) **D***Cystowithiusflorezi* sp. nov. (holotype, ICN-Aps-07) **E, F***Oligowithiusachagua* sp. nov. **E** male paratype (ICN-Aps-388) **F** male holotype (ICN-Aps-388). Scale bars: 0.1 mm (**A–C, E, F**); 0.05 mm (**D**).

***Abdomen***: all tergites divided with a medial suture, with a row of spots in the middle of each hemitergite; sternites entire. Tergal chaetotaxy: ♂, 8: 9: 9: 10: 13: 13: 16: 16: 18: 12: 10 (including two tactile setae): 2; all setae foliate. Sternal chaetotaxy: ♂, 15: (2) 11 (2): (2) 13 (2): 16: 14: 16 + 21 *gls*: 13 + 86 *gls*: 11 + 2 *gls*: 10 (including two tactile setae): 8 (including two tactile setae and some dentate): 2; sternites VII and VIII of ♂ with patches of glandular setae (Fig. 9B); setae uniseriate and acuminate; ♂ without paired invaginations on anterior margins of sternites.

***Genitalia***: see Romero-Ortiz and Sarmiento 2021 as “*Parawithius* msp1”.

##### Etymology.

This species is named after bromeliad plants, due to its close association with them. Most of the specimens were collected by sifting bromeliads on white sheets.

#### ﻿Genus Oligowithius

Taxon classificationAnimaliaPseudoscorpionesWithiidae

Beier, 1936 stat. nov.

CF1D45D1-0521-5BE7-83F7-9FBD75D09B3A

Dolichowithius (Oligowithius) Beier, 1936: 447.

##### Type species.

Dolichowithius (Oligowithius) abnormis Beier, 1936, by monotypy.

##### Diagnosis.

The genus *Oligowithius* can be distinguished from other Neotropical withiid genera by the presence of a patch of glandular setae on sternites VII and VIII either in one or two small circular patches in the middle of the sternites, the distal position of the tactile setae on tarsus IV, the presence of two non-corneate eyes, and the trichobothria *it* and *isb* located far apart. Moreover, the male genitalia are characterized by a pair of lateral apodemes that are not merged with the dorsal apodemes (Fig. 11).

##### Remarks.

Beier (1936) described four new species of pseudoscorpions from the Caribbean islands of Bonaire, Curaçao and Aruba, and from Venezuela. Among them was Dolichowithius (Oligowithius) abnormis Beier, 1936 which represented the type species of the newly described subgenus Oligowithius Beier, 1936. Unfortunately, the description was very short and the justification for the subgenus was limited. The holotype and only known specimen of D. (O.) abnormis cannot be traced in Naturalis Center, Amsterdam (Dr Bram van der BIjl, in litt. 10 July 2019) or the Naturhistorisches Museum, Vienna (Dr Christoph Hörweg, in litt. 9 July 2019), which renders the status of the species and subgenus difficult to assess. We have studied three specimens of an unusual, small withiid from Colombia that closely resembles D. (O.) abnormis in the morphology of the male glandular setae and in the positions of the trichobothria. We found sufficient differences between these specimens and the description of *D.abnormis* to consider it as a new species. We also found sufficient differences between the new species from Colombia and species of *Dolichowithius* to warrant the elevation of *Oligowithius* to full genus level. The differences between *Dolichowithius* and *Oligowithius* are as follows: patch of glandular setae on sternites VII and VIII in *Oligowithius* and on sternites VII–IX in *Dolichowithius*, trichobothria *isb* and *it* far apart in *Oligowithius* and close together in *Dolichowithius*, and the male genitalia with a pair of lateral apodemes that are not merged with the dorsal apodemes in *Oligowithius*, while they are merged in *Dolichowithius*.

#### ﻿ Oligowithius abnormis

Taxon classificationAnimaliaPseudoscorpionesWithiidae

(Beier, 1936) comb. nov.

4C2C34E4-A0AD-58CA-BB53-55A93691BFD4

Dolichowithius (Oligowithius) abnormis Beier, 1936: 446–447, fig. 4.

##### Remarks.

With the recognition of *Oligowithius* as a distinct genus, D. (O.) abnormis is here transferred to the genus *Oligowithius*.

#### ﻿ Oligowithius achagua sp. nov.

Taxon classificationAnimaliaPseudoscorpionesWithiidae

51B6BA65-9882-5A12-BB5F-24F108170BDD

https://zoobank.org/AF485E46-4657-4F22-8462-6CEFDB467C5C

**Figure 10.**
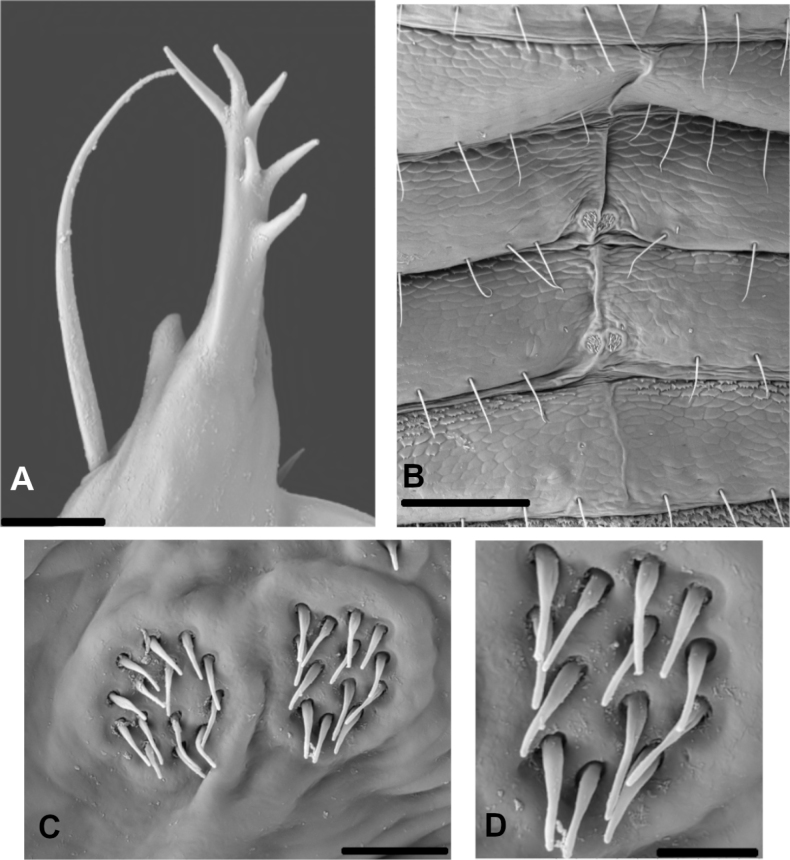
*Oligowithiusachagua* sp. nov. (male paratype, ICN-APs-388) **A** right galea **B** sternites VII–VII **C** patch of glandular setae on sternite VIII **D** detail of the glandular setae on sternite VIII. Scale bars: 100 µm (**A, B**); 10 µm (**C**); 5 µm (**D**).

**Figure 11.**
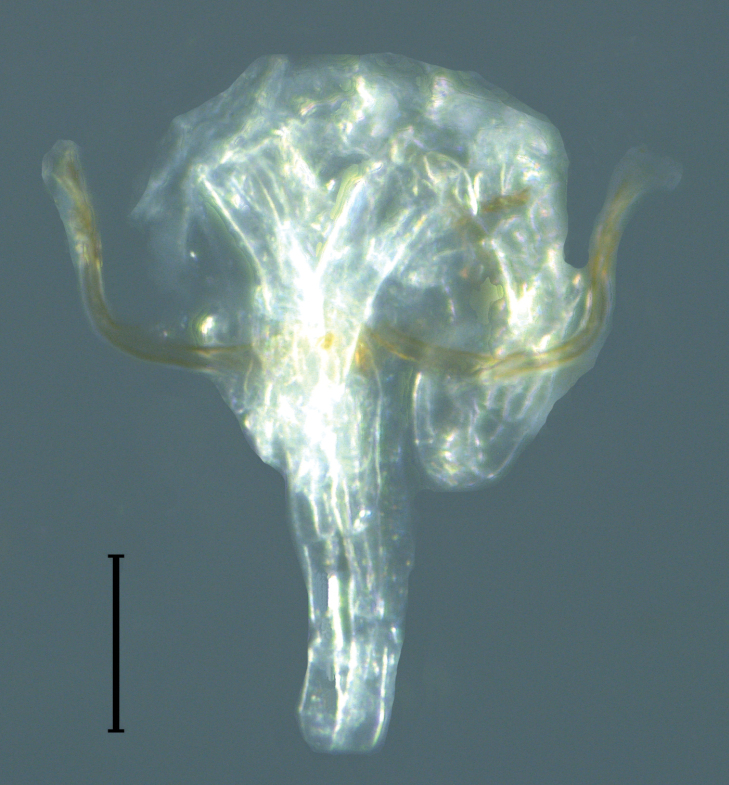
Light micrograph of male genitalia of *Oligowithiusachagua* sp. nov. (holotype, ICN-Aps-388). Scale bar: 100 µm.

Figs 1D , 2 , 8 , 9E, F , 10 , 11

##### Material examined.

***Holotype*.** Colombia • ♂; Meta, Puerto Gaitán, Carimagua; 160 m; 22 Apr. 2012; D. Martinez leg.; estuary on the eastern plains; ICN-APs-388.

***Paratypes*.** Colombia • 1 ♂ and 1 ♀; Meta, same data as the holotype.

##### Diagnosis.

*Oligowithiusachagua* sp. nov. differs from *O.abnormis* by its smaller size (1.7 mm vs 2.18 mm in *O.achagua*), more slender patella in *O.achagua* (3.05× longer than broad) than in *O.abnormis* (2.74×), and a stouter chela in *O.abnormis* (3.70× longer than broad) than in *O.achagua* (4.05× longer than broad).

##### Description.

**Adults.** Color: yellowish brown; carapace and pedipalp reddish brown; legs yellowish, paler than the abdomen, darker in the edges; carapace metazone without paired pale spots (Fig. 1D).

***Dimensions*** (mm): male: holotype (followed by male paratype): body length 2.18 (2.10). Pedipalps: trochanter 0.33/0.16 (0.27/0.15), femur 0.58/0.15 (0.54/0.15), patella 0.54/0.18 (0.50/0.16), chela (with pedicel) 0.94/0.23 (0.88/0.22), chela (without pedicel) 0.88 (0.84), hand (without pedicel) length 0.48 (0.43), movable finger length 0.45 (0.43). Chelicera 0.19, movable finger length 0.16. Carapace 0.74/0.58 (0.68/0.56) (width at medial area); eyespots diameter 0.08. Leg I: femur 0.14/0.14, patella 0.26/0.14, tibia 0.26/0.08, tarsus 0.26/0.06. Leg IV: femur + patella 0.47/0.16, tibia 0.34/0.11, tarsus 0.33/0.06, TS 0.76.

**Female**: paratype: body length 2.36. Pedipalps: trochanter 0.28/0.16, femur 0.54/0.15, patella 0.52/0.18, chela (with pedicel) 0.94/0.25, chela (without pedicel) 0.88, hand (without pedicel) length 0.48, movable finger length 0.44. Carapace 0.73/0.58.

***Carapace*** (Fig. 8A): 1.28 (1.20) (♂), 1.07 (♀) × longer than broad; posterior lateral margins slightly widened; with two non-corneate eyes; with 55 (♂) setae, including 2 (♂) near anterior margin, 11 (♂) near posterior margin, 21 in the medial zone and 23 in the anterior region; with two distinct furrows; posterior furrow slightly closer to posterior carapace margin than to median furrow (Fig. 8A).

***Chelicera*** (Fig. 8B): with five setae on hand, *sb* missing, *b* slightly denticulate, all others acuminate; movable finger with one subdistal seta; galea of male broken in holotype, galea with multiple rami in paratype (Fig. 10A); rallum of four blades, the most distal blade with several serrations on leading edge, other blades smooth (Fig. 8E); two lyrifissures on dorsal side.

***Pedipalp*** (Fig. 8C, H): trochanter, femur, patella and dorsal chelal hand granulate, ventral chelal hand and fingers smooth; dorsal setae clavate and denticulate; trochanter 2.05× (1.79) (♂), 1.75× (♀), femur 3.79× (3.58) (♂), 3.53× (♀), patella 3.05× (3.10) (♂), 2.95× (♀), chela (with pedicel) 4.05× (4.07) (♂), 3.79× (♀), chela (without pedicel) 3.79× (3.89) (♂), 3.55× (♀), hand 2.07× (2.0) (♂), 1.94× (♀)longer than broad, movable finger 0.93× (1.00) (♂), 0.92× (♀)longer than hand. Fixed chelal finger with eight trichobothria, movable chelal finger with four trichobothria (Fig. 8H): *eb* and *esb* situated basally, as well as *ib* and *ist*; *est* situated midway between *it* and *isb* in the middle of the finger, *et* close to fingertip; *b* and *sb* situated near one another, *st* located midway between *sb* and *t*, *t* parallel to *it*. Venom apparatus not visible, venom ducts not visible in ♂; nodus ramosus not visible. Retrolateral margin of fixed finger with two double sense-spots (Fig. 8H) situated midway between *esb* and *est*, movable finger with one double sense-spot close to *sb*. Chelal teeth rounded; fixed finger with 40 (♂) teeth; movable finger with 43 (♂) teeth; accessory teeth absent.

***Coxal region***: coxal chaetotaxy: ♂, 6: 7: 9: 18; maxilla with 19 setae including two apical setae and one very small internal, sub-oral seta; median maxillary lyrifissure medial-anterior in position, posterior lyrifissure present.

***Legs*** (Fig. 8F, G): junction between femora and patellae I and II parallel, junction in legs III and IV oblique; tarsal tactile seta of leg IV situated distally, 0.76 (♂) of tarsus length (Fig. 8G); arolium slightly shorter than claws. Ratios: leg I: femur 1×, patella 1.94×, tibia 3.30×, tarsus 4.72× deeper than broad; Leg IV: femur + patella 2.85×, tibia 3.00×, tarsus 5.13× deeper than broad.

***Abdomen*** (Fig. 8D): first four tergites entire, the others with faint medial suture. Tergal chaetotaxy: ♂, 12: 14: 13: 18: 18: 19: 19: 18: 19: 15: 6: 2, 2; all setae foliate; mostly uniseriate but some tergites with a few setae placed anteriorly. All sternites entire, except for the last three with a faint medial suture. Sternal chaetotaxy: ♂, 10: (1) 6 (1): (1) 8 (1): 14: 12: 8 + 13/13 *gls*: 8 + 18/16 *gls*: 8: 8: 6 (including two clavate setae): 2; sternites VII–VIII of ♂ with two small patch circles of glandular setae each (Figs 9F, 10B–D), paratypes with one circular patch of glandular setae each (Fig. 9E); setae uniseriate and acuminate; ♂ without paired invaginations on anterior margins of sternites.

***Genitalia***: simple structure with most of the components extremely reduced to chitinized lateral apodemes that do not merge with the dorsal apodemes. The level of sclerotization of the ejaculatory channel is weak; however, it is projected from the lateral apodemes, which allows us to classify *Oligowithius* as a Cacodemoniini (Fig. 11).

##### Etymology.

This species is named after the indigenous people, original inhabitants of the Meta, Vichada and the Venezuelan Llanos, the Achaguas. The name should be treated as a noun in apposition.

## ﻿Discussion

In Withiidae, the presence of patches of abdominal glandular setae, especially in males, have been used as a diagnostic character since Weygoldt (1970) first elevated the Withiinae to family level. However, some taxa attributed to the family lack glandular setae, i.e., *Protowithius* Beier, 1955, *Juxtachelifer* Hoff, 1956, and *Termitowithius* Muchmore (Hoff 1946; Beier 1955; Muchmore 1990; Harvey 2015). The arrangement of the patches of glandular setae has often been used to define withiid genera, but without a phylogenetic framework in which to test whether these patterns validly define monophyletic clades. Likewise, trichobothrial patterns seem to provide additional support for some genera, especially when combined with glandular setal patterns and other characters such as the presence or position of tactile setae on the posterior tarsi.

Male genitalia need to be addressed when defining withiid genera. As shown by Mahnert (1975) and Romero-Ortiz and Sarmiento (2021), there is morphological correspondence with genera, and Harvey (2004) suggested that the triangular dorsal apodeme shape found in many withiids likely represented a monophyletic group, the Cacodemoniini. Romero-Ortiz and Sarmiento (2021) were able to define individual Neotropical genera using the morphology of the male genitalia, which is here extended to *Oligowithius*, which is clearly defined based on its genitalia, among other characters. In future, an assessment of the female genitalia may provide further diagnostic features.

Finally, we are aware that a fully resolved phylogeny is needed to support the new clades and the assessment of the old ones; however, as for the new genus *Paciwithius* and for the new genus rank of *Oligowithius*, we presented a unique and exclusive combination of morphological characters that allow us to conclude and support the taxonomic changes presented in this work.

## Supplementary Material

XML Treatment for Paciwithius

XML Treatment for Paciwithius chimbilacus

XML Treatment for Paciwithius valduparensis

XML Treatment for Cystowithius

XML Treatment for Cystowithius florezi

XML Treatment for Cystowithius ankeri

XML Treatment for Parawithius

XML Treatment for Parawithius bromelicola

XML Treatment for Oligowithius

XML Treatment for Oligowithius abnormis

XML Treatment for Oligowithius achagua

## ﻿Additional information

### Author ORCIDs

Catalina Romero-Ortiz https://orcid.org/0000-0001-8939-7814

Carlos E. Sarmiento https://orcid.org/0000-0003-4012-8108

Mark S. Harvey https://orcid.org/0000-0003-1482-0109

### Data availability

All of the data that support the findings of this study are available in the main text.
